# Social Expectations Associated With Adolescent Sexual and Reproductive Health: A Qualitative Study of Tay Adolescent Girls and Their Female Carers in Vietnam

**DOI:** 10.1002/hpja.70250

**Published:** 2026-10-11

**Authors:** Lia Jacqueline Burns, Hannah Pitt, Thuy Doan To Mai, Minh Duc Pham, Peter Azzopardi, Samantha Thomas

**Affiliations:** ^1^ Institute for Health Transformation, School of Health and Social Development Deakin University Geelong Victoria Australia; ^2^ Global Adolescent Health Group Burnet Institute Melbourne Victoria Australia; ^3^ School of Population and Global Health University of Melbourne Southbank Victoria Australia; ^4^ Global Adolescent Health, Murdoch Children's Research Institute and Department of Paediatrics University of Melbourne Parkville Victoria Australia; ^5^ School of Public Health and Preventive Medicine, Monash University Melbourne Victoria Australia; ^6^ The Kids Research Institute Australia, Nedlands, Western Australia, and the Australian National University Canberra Australia

**Keywords:** adolescent girls, female carers, sexual and reproductive health, social expectations, Vietnam

## Abstract

**Issue Addressed:**

This study explored the experience and perceptions of Tay adolescent girls and their female carers in Northern Vietnam related to the dynamics of social expectations of adolescent sexual and reproductive health.

**Methods:**

Critical qualitative inquiry using a ‘Big Q’ experiential approach focussed on subjective experiences. Face‐to‐face interviews were conducted with 20 adolescent girls (16–18 years old) and 20 of their female carers from the Tay ethnic community in Vietnam.

**Results:**

First, social expectations of adolescent Tay girls to uphold traditional standards of femininity and womanhood were shared intergenerationally. Second, this generation of adolescent Tay girls openly challenged social expectations related to their sexual development. Third, many adolescent Tay girls diverged from expectations in relation to their sexuality and demonstrated increased agency and a desire for social change.

**Conclusion:**

This study found that social expectations surrounding adolescent girls’ sexuality were shared across generations, yet perceptions of female sexuality were beginning to shift. This study recommends: formative research on adolescent sexual and reproductive health must consider prevailing social norms; increased momentum and investment for national and local programs that support gender inequities; to mandate and fund local services to increase the knowledge and skills of older generations of women to support adolescent sexual and reproductive health.

**So What?:**

Applying contextually specific data on the different profiles of social norms in a community will improve the impact of health promotion for adolescent sexual and reproductive health.

## 1 Introduction

Global action to address sexual and reproductive health (SRH) inequities for adolescent girls (10–19 years old) remains a priority [1]. Sustainable Development Goal 3.7 addresses SRH and has specific indicators that relate to adolescent girls [2]. The United Nations Population Development Fund [3] defines SRH as a state of complete physical, mental and social well‐being in all matters related to the reproductive system. This state should occur in the absence of disease and illness, with universal access to reliable information, education, counselling and health services. In the last decade, the Global Strategy for Women's, Children's, and Adolescent's Health 2016–2030 [4] and other investments have contributed to significant improvements in SRH outcomes for adolescent girls. Child marriage, adolescent pregnancy and childbirth have declined in many settings with an increase in the uptake of modern contraception [5]. However, persisting inequities exposed during Covid‐19 have highlighted that socio‐cultural norms remain one of the major barriers to adolescent girls achieving equitable SRH outcomes across the Asia Pacific region [6]. Currently 147 million adolescent girls (10–19 years) live in diverse geographical regions and socio‐cultural environments across the Asia Pacific region, where social norms related to female sexuality result in restrictive social expectations for adolescent girls in the family and community [7]. Social norms are the informal rules that guide how adolescent girls are expected to behave in social groups [8]. For adolescent girls these are varied and specific to historical, geographical and socio‐cultural contexts of where they are born and live, with family also being a major influence on how they experience their sexual development [9]. The implications of context for adolescent sexual and reproductive health (ASRH) in this scenario are fundamental to the realisation of equitable or inequitable SRH outcomes for adolescent girls [10]. Poor SRH outcomes for adolescent girls can include early pregnancy, unsafe abortion, sexually transmitted diseases, HIV and mental health issues [2]. Importantly, while poor SRH outcomes often influence the future of the adolescent girl, evidence demonstrates there is also an impact on the health of future generations [11].

### 1.1 Social Expectations and the Sexual and Reproductive Health of Adolescent Girls in Vietnam

Since the 1980's, Vietnam has experienced dynamic economic and socio‐cultural change with a major shift in how the period of adolescence is perceived and experienced, especially for adolescent girls [12]. Now recognised as a key developmental period in the human life cycle, 7 million adolescent girls (10–19 years) across Vietnam [13] have increasing opportunities through globalisation and international integration to migrate for higher education and economic opportunities [14]. Furthermore, wide coverage and uptake of the internet [15] and the digital transformation of health [16] are exposing young people to different ways of living. Research from Vietnam has found that social expectations for adolescent girls to fulfil traditional roles in the family and community do influence socio‐economic opportunities and SRH outcomes [17]. However, examination of social roles in Vietnam shows they are shifting, with adolescent girls and women transcending traditional ideas about their role in family and society [18].

Concurrently, Vietnam is still perceived to be a conservative culture in many ways. Strongly influenced by Confucianism, there are clear social expectations for females and males in the family and society [18]. In Vietnam, these expectations maintain the social structure and often perpetuate gender inequities. This starts early in life, as son preference at birth is still common, with a birth sex ratio of 111.5 males per 100 female births [19]. There is widespread acceptance that women will give birth to one or more sons for the family, resulting in social expectations for marriage and childbearing for female children prioritised across all socio‐economic strata in Vietnam [20]. For adolescent girls, meeting the expectation of their role as wife and mother often requires remaining chaste before marriage to maintain their own and familial reputations [21]. Consequently, the expectation that adolescent girls need access to SRH care is limited, reducing the demand for reliable SRH education and services [22]. However, national and community level data in Vietnam currently shows an increase in adolescent sexual activity outside of marriage [23], with high rates of adolescent pregnancy and abortion [24].

### 1.2 Social Expectations and the Sexual and Reproductive Health of Tay Adolescent Girls

The Tay ethnic minority group (1.85 million people) [25] are 2% of the total population of Vietnam and are the largest ethnic minority group [26]. The Tay live predominantly in the northern mountainous regions of Vietnam, where there are limited options for health and education services [27]. While the qualitative data on Tay adolescent girls is limited, understanding of how they experience health inequities is increasing. National level surveys show high rates of induced abortion in Tay headed households [24] and are consistent with community level qualitative data that shows Tay adolescent girls experience stigma related to sexuality with limited understanding of how to manage their fertility [28]. Such SRH outcomes are often attributed to restrictive socio‐cultural norms related to female health and sexuality, which often occurs in patriarchal societies [29]. Within the Tay ethnic group, patrilineal traditions often position men as the main household decision‑makers, while women face strong social expectations to manage domestic and caregiving roles [30]. The implication of this ideology strongly influences awareness and behaviours of women and adolescent girls, inhibiting development of their confidence to engage with social services, limiting opportunities and perhaps increasing developmental risks [30].

### 1.3 Social Norms Theory and Social Expectations That Impact Adolescent Sexual and Reproductive Health

When considering the social influences on ASRH, the research is clear that social norms shape expectations of adolescent sexuality [31] and needs to be understood in two ways: (i) injunctive norms, what people think adolescents should do, and (ii) descriptive norms, what adolescents actually do [32]. Research shows that adolescent health behaviours are influenced by the injunctive norms shared by important social networks, such as peer and family, known as reference groups [33]. The idea that social expectations are constructed, understood and disseminated among group members through communication [34] underpins how social norms and social expectations function intergenerationally. Lapinski and Rimal [33] state that communication processes within groups highlight how social expectations are formed, transmitted and modified among members of social groups. Exploring how adolescent girls and their female carers communicate about social expectations in proximal settings and how descriptive norms about SRH are changing, may identify those who face unique challenges in particular social settings. Equally, exploring the dynamics of social expectations of adolescent girls could also identify opportunities to engage with organic change.

### 1.4 Knowledge Gap

Globally, social norms related to SRH have been explored significantly more than how social expectations influence SRH for adolescent girls. In Vietnam, qualitative evidence on the lived experience of Tay adolescent girls and their female carers related to SRH is also limited [35]. Existing national‐level quantitative data on Tay communities are growing, but qualitative data exploring health issues often have small data sets that combine ethnic minority groups together, including when exploring the experiences of adolescent girls.

## 2 Methods

### 2.1 Study Setting

The study was conducted across three communes in Thach An District, Cao Bang Province in Northern Vietnam. Approximately half a million people live in Cao Bang Province, consisting of various ethnic minority groups, of which the majority live in rural settings. The Tay ethnic minority group are 40% of the total population of Cao Bang [27]. Cao Bang Province is characterised by mountainous terrain, making travel difficult and costly, particularly during the wet season. Much of the rural population experiences long travel times to health facilities and relies on semi‐boarding schools for access to education for children aged 8 years and above [27].

### 2.2 Study Design

The data presented in this paper was part of a broader study following a qualitative exploratory design to understand the intergenerational experience of social norms related to the SRH of Tay adolescent girls and their female carers. This paper focuses on aspects of the interview where Tay adolescent girls and their female carers shared perceptions and experiences of social expectations impacting SRH and how these might be changing. This work was guided by three research questions: (1) what are the social expectations influencing the SRH of Tay adolescent girls?; (2) how are the social expectations related to SRH perceived by Tay adolescent girls?; (3) are social expectations related to SRH changing in Tay communities? The study applied a ‘Big Q’ experiential approach to reflexive thematic analysis (RTA) [36]. Members of the research team (four women, two men) were public health researchers from Australia and Vietnam with expertise in ASRH and advocacy, and health promotion and the determinants of health. Four members of the research team had previous or current experience working in Vietnam. An advisory group was established from civil society organisations in Vietnam and Australia, who had direct experience working with Tay communities in Vietnam. The advisory group was established to guide the project with representation from ChildFund Vietnam, ChildFund Australia and Centre for Creative Initiatives in Health and Population. The group guided overall study design, development of research questions, technical relevance, focus and cultural considerations. Vietnamese members of the group provided specific technical, cultural and linguistic advice to the research team, feedback on study materials, interpretation and translation support, connections to local authorities for travel permissions and support in the field and hold co‐authorship on published outputs from this study.

### 2.3 Sample, Recruitment and Ethical Considerations

To participate in the study, adolescent girls were required to be between 16 and 18 years old and identify as Tay, and female carers were required to be undertaking a carer’s role within the family. Tay adolescent girls and their female carers were recruited through a commune level household register provided by Thach An District authorities. We sought to recruit adolescent girls and their female carers in an existing relationship from the same Tay‐headed household. Invitations were distributed to 45 Tay‐headed households by local health authorities, supported by the advisory group. Follow‐up phone calls to participants were made by health workers, who facilitated times for the interviews.

A total of 20 adolescent girls and 20 female carers registered to participate, providing highly specific characteristics related to the research questions and rich interview dialogue resulting in sufficient information power [37]. There were two consent processes to complete prior to the interview: (1) the Government of Vietnam Personal Data Collection form applicable in circumstances where personal data is being collected, and (2) the study‐specific consent form. Before the interview, participants were taken through the consent process again. The mandatory Government of Vietnam Personal Data Collection Form was discussed and agreed to first, and then the study consent form was discussed and signed. To further ensure the adolescent girls were comfortable with participating, they were asked to verbally reiterate consent before the interview commenced. The study was approved by a University Human Research Ethics Committee (HREC 2024‐016) and an Internal Review Board, Vietnam (IRB00012062). All participants provided consent to participate in the audio recorded interview.

### 2.4 Data Collection

Interpreter assisted 60 min in‐depth interviews with 40 individuals were conducted separately and audio recorded, providing exploration of experience and perspectives [38]. Two individuals were interviewed via zoom from Australia in early August 2024, to test comprehension of the translated interview schedule and the interpretation process for efficient and cohesive bilingual interviews. No changes were made to the semi‐structured, translated interview schedule. Interviews with 38 individuals were conducted in Vietnam in August 2024. Interviews were conducted in a private room at commune and district level health facilities by the first and third author. The third author led the interviews in Vietnamese, providing a combination of verbatim and summary interpretation to the first author during the interview. The first author provided prompts and follow‐up questions in English, which were interpreted into Vietnamese by the third author. The female carer was interviewed first, followed by the adolescent girl to ensure privacy and that information would not be shared. Participants were provided with a VND 150 000 (AUD10) phone card as a token of appreciation for their time and contribution.

Broad interview topics addressed socio‐demographic factors, perspectives and experiences of expected social roles, intergenerational communication, social expectations and questions about SRH. There were slight variations in interview questions to ensure they were appropriate for the adolescent girl or the female carer. Interview questions related to the social expectations of SRH are provided, see Supporting Information.

### 2.5 Data Analysis

The six steps of RTA were used to engage with the first authors' subjectivity in co‐constructing knowledge, and reporting was guided by RTA reporting guidelines [39]. The first and third authors debriefed daily after data collection to discuss aspects of the interpretation process. The first author commenced familiarisation (Step 1) with manual transcription of the English language interpretation from each audio file. The third author reviewed and quality assured all transcriptions and interpretation, discussing meaning and intent with the first author. Microsoft Excel spreadsheets were used to manage qualitative data and were available to the research team members. The first author reviewed the files, took notes and coded (Step 2) data that was consistent with research questions related to social expectations of SRH and guided by concepts of social norms theory. The social norms theory concepts of descriptive and injunctive norms [32], communication patterns [40] and reference groups [33] informed the analytic orientation by sensitising the analysis to accounts of perceived social expectations and common practice, approval and disapproval related to SRH. Research team meetings considered the initial coding, and the first author proceeded to develop the themes (Step 3) from inductive perspectives shared by the participants and informed deductively from her professional experience, rather than through a fixed deductive coding framework. All authors considered the meaning of the transcribed summaries and quotes in the context of the broader interview and challenged any potential stretching of meaning. The themes were reviewed (Step 4) and reflected on by all authors before finalising the constructed themes (Step 5). All authors agreed on quotes that represented the thematic analysis. Three themes were agreed upon which guided the final iterative write up process (Step 6) with ongoing reflection and input from all authors. The logistics of providing feedback to participants in this study are currently being negotiated by the advisory group and findings will be shared through a translated summary of this publication.

## 3 Results

### 3.1 General Characteristics

Table 1 presents the general characteristics of participants. Overall, 40 participants joined individual interviews. All adolescent girls identified as Tay, and female carers identified as Tay (*n* = 12), Nung (*n* = 7), and Kinh (*n* = 1). The majority of female carers were mothers to the girls (*n* = 18) and were in paid employment (*n* = 17). All adolescent girls (*n* = 20) were completing or had completed high school and *n* = 8 female carers had completed high school with *n* = 7 completing tertiary education.

**TABLE 1:** General characteristics of participants.

Characteristics table		
	Girls (*n* = 20)	Female carers (*n* = 20)
Age		
16	7	
17	8	
18	5	
18 to 25	—	2
26 to 35	—	—
36 to 45	—	8
46 to 56	—	10
Ethnic group		
Tay	20	12
Nung	—	7
Kinh	—	1
Religion/spiritual belief		
Ancestor worship	20	20
Relationship status		
Married	—	17
Widowed	—	2
Boyfriend	4	—
Single	16	1
Education		
Never attending school	—	1
Primary school	—	1
Secondary school	—	1
High school	20	7
Intermediate degree	—	3
College	—	3
University	—	4
Relationship of female carer to girl		
Mother		18
Sister‐in‐law		1
Sister		1
Female carers employment		
Home duties		2
Farmer		6
Teacher		4
Health worker		4
Retail and sales		2
Factory worker		1
Retired		1

Three themes were constructed from the qualitative data. The first theme showed that certain social expectations related to the SRH of adolescent girls were being passed intergenerationally between women. The second theme demonstrated how adolescent girls were perceiving expectations of SRH issues, and the third theme illustrated how adolescent girls were starting to respond differently to social expectations related to their sexual development.

### 3.2 Theme 1: Traditional Standards of Social Expectations Related to the SRH of Tay Adolescent Girls Are Perceived to Be Transmitted Intergenerationally

Overall, participants shared that discussing SRH topics was sensitive and not encouraged. However, social expectations of adolescent girls to exhibit cultural standards of femininity and chastity were being passed down intergenerationally. Adolescent girls shared that parents expected them to maintain modesty and/or to protect themselves from sexual harassment. They shared that parents told them that if they wore the wrong clothes, they would be to blame for any harassment. Adolescent girls and female carers recalled that there were social expectations for females to avoid pre‐marital sex. A few adolescent girls shared that some people think ‘keeping your virginity for your husband is important’. Both adolescent girls and female carers shared the perception that these social expectations were not applied to adolescent boys. While female carers told adolescent girls to avoid pre‐marital sex, they also reminded them of the inequity between expectations, with one female carer telling an adolescent girl that ‘sex is different for a boy’. However, one adolescent girl felt that boys also had a responsibility to prevent pregnancy.

… all the family needs to learn, not just teach girls to prevent pregnancy but teach boys to be responsible too. Pair 15, Adolescent Girl, Age 16

There was a strong social expectation that adolescent girls would definitely get married and have children. Most participants considered that marriage would happen when the adolescent girl had finished school or university. One adolescent girl shared that her aunt could not understand why girls would not get married, warning that people would think you are a ‘poisonous girl’ without children. Consistent with their experience as young women, female carers shared their expectation that adolescent girls would learn how to do household chores, know how to prepare meals and take care of the family. In the expectation that adolescent girls were not sexually active prior to marriage, some female carers shared that adolescent girls only needed to be aware of SRH information and services when they were ready to be married. Others held a perception that only married mothers could access these services anyway. Some adolescent girls held similar views and believed it would only be necessary for them to receive comprehensive SRH when preparing for marriage.

… in the future it will be necessary to receive more SRH information, before marriage. Pair 11, Adolescent Girl, Age 16

Son preference and the higher status of boys in the family were topics mentioned by both adolescent girls and female carers. Participants perceived a clear gender hierarchy in which the birth of boys was preferred. Consequently, adolescents boys had fewer social expectations of them, held great priority in the family and had more sexual freedoms than adolescent girls. Adolescent girls shared their perception that boys were preferred because when girls get married they ‘won't return home anymore’ to care for the family. Female carers gave the perception that they would not address sexuality issues or behaviours with their adolescent sons. For adolescent girls, the expectation to bear male children placed them under considerable pressure from family and the wider community. One adolescent girl reported feeling ‘mistreated’ because of the social expectation to have a male child. Female carers also felt the burden of being expected to have boy children, with one participant reporting that her husband was sad because they only had two girls. However, one female carer shared that although its ‘from the past’ she knows families that prefer sons and where girls do all the chores.

Girls have to do chores, and the boys can just relax. It's from the past. Some of (my) friend's families still have these son preference, the girls have to do all the chores. Pair 2, Female Carer, Age 18

### 3.3 Theme 2: Tay Adolescent Girls Are Challenging the Intergenerational Social Expectations of SRH

Adolescent girls understood the social norms shared by their female carers, yet had begun to question and challenge the nature of these expectations. All participants shared that they had experienced difficulties in talking about SRH subjects and mostly avoided engaging on these topics within the matrilineal line, including female carers who were also trained health workers. However, adolescent girls expressed frustration with the sensitive nature of SRH topics, sharing the wish that adults would ‘normalise these topics … (and) not make it a sensitive topic’ Pair 15, Adolescent girl, Age 16. Female carers shared their experience of limited access to SRH information or education as younger women and gave the perception that they did not expect to share this information with the younger generation either. Although, a female carer shared that there was a social expectation that female family members were responsible for educating the younger generation about SRH. Shared stories by both adolescent girls and female carers gave the perception that they felt they had to learn about SRH themselves. However, adolescent girls said that they would like to talk about these issues more and have family members share their knowledge and experience.

(I) would like my mum and sister to share more information. They don't share much. Pair 4, Adolescent Girl, Age 16

Adolescent girls gave the perception that they felt controlled by the social expectations currently passing between generations of women. Female carers shared expectations that adolescent girls should conduct themselves appropriately, so as to avoid sexual harassment, sexual activity and pregnancy before marriage. Aunts, grandmothers, female neighbours and teachers all had similar expectations for the adolescent girl. Female carers shared that their mothers also had similar expectations of them to avoid sex and pregnancy before marriage. One adolescent girl shared that when her peer at school became pregnant, her parents became stricter and did not allow her to go out at nighttime anymore and that such parental control was ‘unreasonable’. Another adolescent girl was concerned that strict control of an adolescent girls’ behaviour and imposed ideas by parents would affect the mental health of an adolescent girl, and she may pass such ideas onto her children.

… how the girls are being treated might affect their mental health because they were raised in a strict environment with imposed ideas; and therefore, they might do the same to their children, like pass their habits onto their children. Pair 2, Adolescent Girl, Age 16

Adolescent girls expressed concern about experiencing shame, fear and stigma in order to meet social expectations of their sexual development. All participants shared views directly or indirectly that adolescent girls should not be sexually active, with shame and fear being instilled in adolescent girls across generations. Some adolescent girls internalised the restrictive attitude to sex, shared a range of emotions such as feeling ‘scared of having sex’, conscious that others would think they were ‘shallow’, or think that they would not enjoy good health if they were sexually active. One adolescent girl shared that she would not have sex with her boyfriend if he asked, because she was afraid. Female carers also reported that their mothers tried to scare them into delaying sexual activity. Adolescent girls were also concerned about the attitudes of adults related to sexuality, with one adolescent girl expressing that she didn't like that her Dad and teacher considered her pregnant school friend to be a burden on society.

… she was a good senior student who got pregnant, the teacher used her as a case study, she looked down on her, like the girl made a mistake and was not well taught and made mistakes. That's what my Dad thinks too. They made mistakes and are bad people and a burden on society. But (I don't think) it's like that. Pair 15, Adolescent girl, Age 16

### 3.4 Theme 3: This Generation of Adolescent Girls Are Responding Differently to Social Expectations Related to SRH

Although social expectations relating to SRH were persisting between generations, there were signs that adolescent girls were beginning to take a different pathway to adulthood in this community. Participants all perceived marriage and childbearing to be an important lifecycle milestone but recognised that socio‐economic changes were bringing more options for this generation of adolescent girls. Adolescent girls and female carers noted that young people were exposed to more information than previous generations due to internet access and use of social media. A decline in arranged marriages and the early marriage law was also cited by many participants as bringing significant change to past traditions. Many adolescent girls gave the perception that they were considering delaying marriage, as they shared their intentions of completing high school, seeking further education, broader life experience and employment. Some female carers reported being happy that adolescent girls now have an easier life compared to when they were growing up. One adolescent girl shared that she had not thought about marriage and wanted to be independent.

(I) haven't even thought about getting married. (I) want to be independent and financially independent and buy a house. Pair 15, Adolescent Girl, Age 16

While social expectations related to SRH were conservative overall, many participants gave the perception that people's views about female sexuality and the sexual behaviour of adolescent girls was changing. Many adolescent girls shared that they felt more curious about sex, and that sex before marriage was becoming more common among their peers. One adolescent girl thought people were becoming more open about their sexual orientation and more comfortable expressing themselves. Two female carers also noted that people were more open minded than before, and shared the view that virginity was now less important than avoiding pregnancy before marriage. One female carer made a comparison to explain a change in the perception of adolescent girls' sexual behaviours over time. This participant shared that her mother spoke to her about menstruation when she was the same age as the adolescent girl, and now she talks to her adolescent girl about protecting herself from pregnancy before marriage and avoiding boys.

(My) mother taught me about menstrual hygiene when (I) was about her age, now I talk to my daughter about protecting yourself and not listening to boys. Pair 1, Female Carer, Age 40

Some participants in this community shared stories that illustrated how this generation of adolescent girls were raising their voice with increasing agency regarding their SRH. Adolescent girls shared that they expected ‘change’ in the way they accessed SRH information and education; were curious about their sexuality and had ideas about how they could be better supported in their sexual development. One adolescent girl gave the perception of hoping for a less conservative community, wanting people to change their mindsets and the way they live. One female carer felt that girls should join activities at the health clinics so they can learn more from the health workers and another felt adolescent girls had ‘the right to participate’ in decision making about health services. Some adolescent girls also expressed interest in joining community health campaigns as volunteers and learning more about SRH issues so they could help others. They also felt that they wanted better SRH education at school and more engagement with health workers.

(I) want people to know and teach young people that these issues happened because of instinctive sexual desire or not being (well) taught (by family or at school) about how to prevent themselves from getting pregnant. Young people should receive CSE when they are in secondary school or even primary school. Pair 15, Adolescent girl, Age 16

## 4 Discussion

The study aimed to explore the perceptions of Tay adolescent girls and their female carers regarding which social expectations of SRH were being passed intergenerationally, how adolescent girls perceived these social expectations and if they were changing. There are three main areas of discussion.

First, there were a range of social expectations of adolescent girls that were being shared intergenerationally. Defined by injunctive norms [32], social expectations set societal and familial standards for how adolescent girls are expected to conduct themselves. Social expectations for adolescent girls to exhibit culturally appropriate traits of femininity and chastity were implied by female carers and other family members. The intergenerational expectations being passed on to adolescent girls included being modest, taking responsibility for avoiding sexual harassment, avoiding pre‐marital sex, getting married and producing one or more sons, and learning to be wife and how to take care of the family. In contrast, adolescent boys were not held to the same standard of conduct, perpetuating gender inequities and double standards across generations. The literature reflects how Confucianism and patriarchy in Vietnam across both urban and rural communities support social hierarchies with different and unequal expectations for females and males [41]. There were more restrictive expectations for females than males related to sexual conduct and clear roles for females within marriage, related to reproduction and household labour force [30]. In this cultural context, the expectation that females will marry and bear sons has been examined widely in the literature, showing that the guiding injunctive and descriptive norms were consistent across all socio‐economic and geographical strata in Vietnam [18, 20]. Research into population policy in Vietnam shows how the implication of this social expectation can undermine women's health and wellbeing through possible coercion and violations of reproductive and bodily autonomy [20]. These findings contribute knowledge to a limited evidence base regarding the life and experience of Tay adolescent girls and their female carers, demonstrating the intersectional challenges in this community of patriarchy, geographic isolation, secondary students living away from family to access education and low health and digital literacy [35]. The experience of Tay adolescent girls and their female carers in this community was consistent with the existing literature on other ethnic groups in Vietnam, suggesting that intergenerational expectations of adolescent girls was contributing to SRH inequities and surpasses factors of ethnicity.

Second, adolescent girls were starting to challenge and question the social expectations related to SRH that were shared by their female carers. As interpersonal communication is the principal channel of how injunctive norms [32] related to social expectations are shared between generations [40], this space is an opportunity to support adults to engage with adolescents on SRH in a more relevant way. A 2023 systematic review of parent‐adolescent SRH communication initiatives demonstrated that evidence‐based SRH information communication interventions were effective in improving engagement between parents and adolescents on SRH and increasing safe sexual behaviour [42]. However, a 2025 systematic review and meta‐analysis noted the lack of comprehensive research on these programs in complex socio‐cultural settings and in contexts where a particular social standard or moral attitude to adolescent sexuality prevails [43]. Consequently, in a dynamic socio‐cultural setting like Vietnam, implementation research into how these initiatives could be adapted to utilise intergenerational shifts in traditional standards within reference groups [33] may lead to increasing the relevance and effectiveness of such approaches. A recent study from Northern Vietnam recommended that civil society organisations have a role to play in contextualising global best practice for local communities, increasing the effectiveness of national policies and resources [44].

Third, there is evidence of some adolescent girls not conforming to shared social expectations and becoming sexually active prior to marriage, in contrast to previous generations. These descriptive norms illustrate social change, where adolescent girls were exploring and acting beyond how they were told they should behave. Adolescent girls were challenging the pathways to traditional marriage, aware of how improved socio‐economic conditions are providing easier lives and more choice for this generation of adolescent girls. Closely associated with changing views on marriage was the increasing sexual activity of adolescent girls and recognition of the inevitable fact that adolescent girls were becoming sexually active earlier and before marriage. Consequently, adolescent girls were showing increasing agency in actively seeking SRH information and expecting social change that normalises SRH. These findings are consistent with literature that explores traditional and modern expectations of adolescent girls in Vietnam [45]; and how the social norms approach to addressing ASRH inequities often focuses on building the empowerment of women and girls [46, 47]. Perhaps identifying the intersection and timing of naturally occurring shifts in injunctive and descriptive norms could be a catalyst for change.

### 4.1 Strengths and Limitations

The engagement of the advisory group was a significant strength of this study and was possible due to the professional relationship between the first author and advisory group members. The first author drew on the expertise of the Vietnamese national members and their knowledge of the Tay community to develop relevant research questions and address linguistic and cultural factors. In this way, the advisory group moderated the consequence of limited access to Tay communities by foreign research teams during study design, which enabled methodological congruence and relevance for the socio‐cultural context.

The research was conducted in Thach An District, Cao Bang Province; therefore, findings from other Tay headed households across Northern Vietnam may differ. The registration process revealed intermarriage in this district, with just over half of the female carers identifying as Tay. The degree to which this influenced the findings was not explored in the study. Interview audio files were not fully translated from Vietnamese to English due to time and resources, but all summary transcriptions were verified and used in the analysis and interpretation process. A selection of translated and transcribed summaries was checked against original audio files to ensure they provided a comprehensive record of the discussion. Although the third author aimed to provide verbatim interpretation during interviews, some real‐time summary interpretation was used to support interview flow and clarify meaning. This was particularly relevant when participants used indirect phrasing, locally specific expressions, or non‐technical terms. To reduce loss of meaning, the third author provided contextual clarification to the research team during interpretation and analysis.

## 5 Conclusion

This study found that social expectations related to SRH were being transferred between women and girls across generations, with adult females perpetuating the messaging regarding socially accepted behaviours for adolescent girls. However, despite the ongoing conservative perspectives and expectations, there were stories of change evident in shifting injunctive and descriptive norms. Adolescent girls were showing more autonomy and agency in making their own decisions about sexual activity and future union. Some female carers recognised this changing social dynamic and wanted to support their daughters. In this community, where there were insufficient ASRH services and external support was rare, social change was happening organically. Further exploration of the dynamics of social change in context will help to find the tipping point of norms change [8]. This study recommended consideration of the following actions: formative research on any aspect of ASRH should include exploration of prevailing injunctive and descriptive SRH norms and social expectations in the community; increased momentum and investment in existing national and local programmes that support gender inequities related to the role of women and girls in society; fund and mandate local services (Women's Union, community public health campaigns) to increase the knowledge and skills of older generations of women to support adolescent girls in their sexual development.

## Funding

This work was supported by an Australian Government Research Training Program awarded to L.J.B.

## Ethics Statement

Ethical approval for this study was obtained from two ethical review boards. Firstly, the study was approved by the Deakin University Human Research Ethics Committee, Victoria, Australia (2024‐016), and then submitted to and approved by the Ethics Committee at the Centre for Creative Initiatives in Population Health, Hanoi, Vietnam. Ethical clearance was obtained in accordance with the Helsinki Declaration https://www.wma.net/policies‐post/wma‐declaration‐of‐helsinki/ and the Australian Code for the Responsible Conduct of Research 2018. Australian Code for the Responsible Conduct of Research 2018 | NHMRC.

## Consent

All participants over 18 years old provided written and verbal consent. Participants under 18 years old provided written and verbal assent and their female carers also provided consent for their participation.

## Conflicts of Interest

The authors declare no conflicts of interest.

## Supporting information


**Data S1:** Interview questions related to the social expectations of sexual and reproductive health.

## Data Availability

The data that support the findings of this study are available on request from the corresponding author. The data are not publicly available due to privacy or ethical restrictions.
