# Supplementary material for: Social Expectations Associated With Adolescent Sexual and Reproductive Health: A Qualitative Study of Tay Adolescent Girls and Their Female Carers in Vietnam
Source: Health Promot J Austr. 2026 Oct 11;37(4):e70250. doi: 10.1002/hpja.70250 (PMC13655298; doi:10.1002/hpja.70250)
Supplement: Supplementary file 1 [file HPJA-37-0-s001.docx]

**Supplementary Material**

**Interview questions related to social expectations of sexual and reproductive health**

| **Tay adolescent girls (n=20)** | **Female carers (n=20)** |
| --- | --- |
| - To what extent have you discussed these sexual and reproductive health terms or related issues with other people? Who do you talk too and what about?   - Are some of these terms more embarrassing or taboo to talk about than others? - What is the most important issue for you regarding the sexual and reproductive health of adolescent girls? - Why is this issue the most important matter to you? How do you feel about this issue?   - Have you discussed this issue with anyone else? - How have you received information or education about this issue? Would you like to learn more? - We have shared some examples of sexual and reproductive health topics. What do you think people in your community expect from girls related to these issues?   - What do you and your friends talk about in relation to expectations related to any of these topics?   - Do you think social expectations are important for girls? Why?  - What do you think your parents or community leaders expect from girls related to some of these issues? Who in your community is the most vocal about how girls should behave?   - What do older women in your life tell you about these social expectations? For example, about using contraception, sex before marriage.   - How does this make you feel?   - Do you think what these women tell you may affect your health? How? - Would you share your experience or that of someone you know who has been affected by the social expectations we have just discussed?   - How do you think this experience affects the health of girls?  - How does this story make you feel?   - Thinking about what we have discussed, how do you think your experience of being a girl is different from that of your mother/older women in your community, when they were your age?   - If not different, what is the same?   - If different, can you give me an example?   - Has your female carer ever shared any experience from when she was young?   - How does this make you feel? - What do you think has changed the most in your community since your mother/older women in the community, were girls? | - What is the difference between what girls/women do with their day and what men do with their day?   - What do you tell your daughter/young girls about her duties?  - What does your husband think about the role of girls in the home?  - What did your female carer share with you about your role when you were a girl?   - What extent have you discussed these sexual and reproductive health terms or related issues with other people? Who do you talk too? - What is the most important issue for you regarding the sexual and reproductive health of adolescent girls?   - What did your mother tell you about this ISSUE when you were young?   - Why is this issue the most important matter to you? How do you feel about this issue? - How have you received information or education about this issue? Can you share your experience? - Did you talk to your daughter about this issue?   - If no, why?   - If yes, what did you share?   - Why is this issue important for your daughter?   - How could she be affected by this issue if she does not know? - We have shared some examples of sexual and reproductive health topics. What do you think people in your community expect from girls related to these topics?   - What social expectation related to SRH would you share with your daughter or younger women? Why?   - Are there greater social expectations for girls related to some of these topics more than others? Why? - What do you think is the purpose of these social expectations?   - Are these beneficial to girls? - Would you should share your experience or that of someone you know who has been affected by the social expectations we have just discussed?   - How does this make you feel? - Thinking about what we have discussed, how do you think your experience of being a girl is different from that of your daughter/other young girls at the same age?   - If not different, why, what is the same?   - If different, can you give me an example? - What do you think has changed the most in your community since you were a girl? |
